# Antibody Biomimetic Material Made of Pyrrole for CA 15-3 and Its Application as Sensing Material in Ion-Selective Electrodes for Potentiometric Detection

**DOI:** 10.3390/bios8010008

**Published:** 2018-01-19

**Authors:** Alexandra R. T. Santos, Felismina T. C. Moreira, Luísa A. Helguero, M. Goreti F. Sales

**Affiliations:** 1 BioMark/CINTESIS@ISEP, Instituto Superior de Engenharia do Instituto Politécnico do Porto, 4249-015 Porto, Portugal; alexandrarsantos@gmail.com (A.R.T.S.); ftcmo@isep.ipp.pt (F.T.C.M.); 2 Departamento de Ciências Médicas, Universidade de Aveiro, 3810-193 Aveiro, Portugal; luisa.helguero@ua.pt

**Keywords:** plastic antibody, molecularly-imprinted polymers, Breast Cancer Antigen, CA 15-3, potentiometry

## Abstract

This work reports a very simple approach for creating a synthetic antibody against any protein of interest and its application in potentiometric transduction. The selected protein was Breast Cancer Antigen (CA 15-3), which is implicated in breast cancer disease and used to follow-up breast cancer patients during treatment. The new material with antibody-like properties was obtained by molecular-imprinting technology, prepared by electropolymerizing pyrrol (Py, 5.0 × 10^−3^ mol/L) around Breast Cancer Antigen (CA 15-3) (100 U/mL) on a fluorine doped tin oxide (FTO) conductive glass support. Cyclic voltammetry was employed for this purpose. All solutions were prepared in 4-(2-Hydroxyethyl)-1-piperazineethanesulfonic acid (HEPES) buffer, of pH 6.5. The biomarker was removed from the imprinted sites by chemical action of ethanol. The biomimetic material was then included in poly vinyl chloride (PVC) plasticized membranes to act as potentiometric ionophore, having or not a lipophilic ionic additive added. The corresponding selective electrodes were evaluated by calibration curves (in buffer and in synthetic serum) and by selectivity testing. The best analytical performance was obtained by selective electrodes including the plastic antibody and no lipophilic additive. The average limits of detection were 1.07 U/mL of CA 15-3, with a linear response from 1.44 to 13.2 U/mL and a cationic slope of 44.5 mV/decade. Overall, the lipophilic additives yielded no advantage to the overall potentiometric performance. The application of the MIP-based electrodes to the analysis of spiked synthetic serum showed precise and accurate results.

## 1. Introduction

Breast cancer is the second most common cancer disease in the world, representing 25% of all cancers. Its early and quick diagnosis is becoming more important within time, because its incidence tends to increase, mostly in women above 45 years old [1,2]. The first approach to a diagnosis in breast cancer is clinical examination and complementary exams such as imaging and biopsy. In the biopsy, the sample collected is analyzed with regard to its estrogen and progesterone receptors, aiming to identify the type of breast cancer. This technique is very informative and to date is the standard of choice. However, it is invasive, has risks to the patient and relatively expensive. This points out the need for new diagnosis methods to assess cancer biomarker expression [3,4].

Today, there are many reports showing the existence of specific biomolecules in biological fluids, such as urine, blood and saliva, which may lead to the non-invasive diagnosis or patient follow-up of many diseases, including breast cancer. Such biomolecules could be regarded as biomarkers when their levels act as disease indicators. These are particularly important when found in biological fluids as they are not present in healthy people, or if present, their levels are altered [3,4,5,6]. Many biomarkers exist in the context of breast cancer, with CA 15-3 taking the lead as standard use to assess patients at several stages of disease development, including the prognosis after surgery or chemotherapy [7,8,9,10]. CA 15-3 is expressed by MUC-1, a glycoprotein that is overexpressed in breast cancer. The typical limiting value of CA 15-3 in clinical context is 30 U/mL which may range 25 to 35 U/mL depending on the exact method employed for its determination [7,8,9,10].

The determination of CA 15-3 in the hospital environment relies mostly on Enzyme-Linked Immunosorbent Assay (ELISA) assays, making use of antibody/antigen interactions. The use of such interactions has also been moved to biosensing technology, about which the most recent works in the field have been resumed later [11,12,13,14,15,16,17,18]. Overall, the use of antibodies ensures good selectivity and sensitivity, but it is also linked to strict storage conditions, limited stability, and high cost. Other methods concurring with ELISA are separative techniques, such as electrophoresis [19], but these are not suited for routine control in point-of-care, due to their inability to perform analyses outside laboratory facilities, and their high cost and environmental impact.

Replacing natural antibodies with biomimetic synthetic materials (also known as plastic antibodies) may avoid the typical limitations of methods using natural antibodies. Plastic antibodies are currently obtained by molecular imprinting technology (also known by molecularly-imprinted polymers (MIPs) [20,21,22]. These materials may also be coupled to several transduction schemes, including potentiometric methods, offering several advantages in terms of cost, rapidity and portability with ion-selective electrodes [23,24].

Obtaining an antibody-like material for CA 15-3 based on molecular imprinting techniques implies the use of experimental conditions that are compatible with such biological material [8,25,26]. In general, CA 15-3 biomarker is a protein with glycoside fragments resulting from an aberrant post-translational glycosylation profile. Thus, polymerization should occur under conditions close to those under physiological environment (mostly regarding pH and ionic strength, to allow retaining its conformation under physiological conditions).

Among the different kind of polymerization processes, electropolymerization is emerging in the literature as a promising alternative to chemical-based procedures [27]. In electropolymerization the polymer grows by an electrical stimulus, thus allowing a high structural control over the growth of the polymer, and enhancing the precision of the process. In addition, electropolymerization requires less reagents, reducing costs and improving the environmental impact of such processes, while reducing the operator intervention and the complexity of the imprinting step [28]. Thus, electropolymerization was selected herein to synthetize the MIP material for CA 15-3, which was done in a solution containing only monomer (as building block of the polymeric structure) and biomarker (as a target molecule).

The monomer selected for this polymerization was pyrrole, because its electrical polymerization is well-known and efficient [29]. The resulting polymer was polypyrrol (PPy), which has been employed several times in molecular imprinting [30,31,32]. In general, electrostatic interactions are easily established between a negatively charged protein and a positively charged polymer. Hydrogen bonding is also likely to occur between these through the N-H bond of the polymer. Yet, the nanostructure of the electropolymerized pyrrol (Py) depends of the electrical conditions set for polymeric growth, varying within nanobelts, nanobricks or nanosheets [29]. As the scanning velocity of the polymeric position increases, the time granted to deposition and dissolution of the polymer is lower, resulting in a decreased level of recrystallization. Considering this, the decrease in the deposition time (faster scanning speed) originates a more porous structure, organized in multiple layers of nanosheets, while the increase of this time (lower scanning-rate,) would result in a denser and more compact nanobelt structure, organized in an additionally aggregated way [29].

Thus, the antibody-like material was obtained by electropolymerizing Py in the presence of the target biomarker, CA 15-3. Different electrochemical conditions regarding scanning-rate were tested to identify the one leading to the most effective plastic antibody. The overall performance of the material was tested by incorporating it as an ionophore in ion-selective electrodes (ISEs), and testing the effect of lipophilic additives with different charges upon the potentiometric response. The overall evaluation and optimization are described next, demonstrating the potentiality of this work to be applied to the analysis of serum samples in point of care (POC).

## 2. Experimental Section

### 2.1. Apparatus

All electromotive force (emf) measures were made in a decimilivoltammeter from Crison (±0.1 mV, µpH 2002) at room temperature, under continuous stirring. This equipment was connected to a homemade 6-way out switch-box, allowing the simultaneous reading of 6 electrodes. The potentiometric cell includes a conductive epoxy-graphite contacting portion with an ion-selective membrane (Table 1) and a KCl/AgCl(s)/Ag electrolyte solution (double junction, Orion).

Fourier transform infrared spectroscopy (FTIR) analysis was made in a Nicolet iS10 FTIR spectrometer, with an attenuated total reflectance (ATR) support with a germanium crystal. Spectra were taken after correction of the background and 32 repeated scans. Raman spectroscopy studies were made in a Thermo Scientific DXR Raman coupled with a confocal and a 532 nm laser. The samples were analyzed with a 0.3 mW laser and a 25 μm slit aperture.

Morphological surface analysis was conducted in a Scanning Electron Microscope (SEM) operating in a vacuum mode, from JEOL, JSM-6010LA (JEOL, Tokyo, Japan).

### 2.2. Reagents

All reagents used were pro-analysis grade, obtained from different sources: Phosphate buffered saline (PBS) from VWR; 4-(2-Hydroxyethyl)-1-piperazineethanesulfonic acid (HEPES), Cancer Antigen CA 15-3, Pyrrol (Py), Interleukin 6 (IL-6) and Serum Cormay HM from Sigma-Aldrich (St. Louis, MO, USA) Poly (vinyl chloride) of high molecular weight (PVC) and Tetrahydrofuran (THF) from Riedel-deHäen (Berlin, Germany); *Tetrakis*(4-chlorophenyl)borate (PClTPB) and 2-Nitrophenyl octyl ether (oNPOE) and Ethanol from Fluka (Bucharest, Romania); Tetraoctylammonium bromide (TOB) from Acros (Ottawa, ON, Canada); Ethylene glycol from TCI (Portland, OR, USA); Sodium hydroxide (NaOH) from Scharlau (Barcelona, Spain); and Carcinoembryonic Antigen (CEA) from Biorbyt (Cambridge, UK). De-ionized water of conductivity <0.1 µS/cm was employed throughout.

### 2.3. Solutions

The stock solution of CA 15-3 was prepared in PBS buffer of pH 7.4 and had a concentration of 100 U/mL. When necessary, standard solutions of lower concentration were obtained by accurate dilution of this stock solution, made in HEPES buffer, of pH 6.3 and 1.0 × 10^−3^ mol/L.

The selectivity study was made by calibrating the electrodes in the presence of a possible interfering species. CEA and IL-6 was selected for this study. CEA had a concentration of 3.0 × 10^−3^ ng/mL and IL-6 a concentration of 1.66 pg/mL.

### 2.4. Synthesis of Antibody-Like Polymers

Before electropolymerization, fluorine doped tin oxide (FTO) glasses were cleaned with ethanol, followed by cyclic voltammetry (CV) electrochemical cleaning. In this, each glass was submerged in a sulfuric acid (0.5 mol/L) and subjected to 15 cycling scans, between −0.2 and +1.6 V, at a scan-rate of 50 mV/s.

Electropolymerization was conducted on the cleaned-FTO surfaces, by applying potential changes ranging from −0.2 to +1.0 V, for 40 cycles, in CV experiments. The scan-rate was varied, and the optimum value selected. The polymerizing solution contained monomer (Py, 5.0 × 10^−3^ mol/L) template (CA 15-3, 100 U/mL) and ethylene glycol (1%), all prepared in PBS buffer, pH 7.4. The resulting polymer was a black film visible on the FTO support (PPy/CA 15-3), which was carefully washed with distilled water and subsequently removed (using a spatula). The polymer obtained was scraped and grounded to powder. The solid collected was insoluble in aqueous solution and incubated in ethanol (1 mL) for 1 h, under constant agitation. Afterwards, the solid was isolated by centrifugation, and dried, at 60 °C in a dry-oven. Subsequently, multiple washes in PBS buffer were performed, in order to remove possible residues of CA 15-3 or unreacted reactants. Finally, the powder was washed with distilled water and dried in a desiccator under a nitrogen atmosphere.

A control material, named non-imprinted polymer (NIP), was prepared similarly in the absence of the template protein, including the protein removal stage.

### 2.5. Selective Membranes Preparation

The CA 15-3 selective membranes combined a polymer, a plasticizer and the ionic sensing material (MIP). In detail, these were prepared by mixing 14.0 mg of PVC, 30.0 mg of oNPOE and 2.3 mg of MIP and NIP materials. In parallel, some membranes were added of 0.28 mg of lipophilic additive: PClTPB, acting as anionic additive, or TOB, acting as cationic additive. The final mixture was intensely stirred until dissolution of the polymeric PVC was achieved and after dispersed in 1.0 mL THF. The resulting membranes were applied on graphite-based solid conductive supports [33] and let dry for 24 h. The electrodes were kept in HEPES buffer when not in use.

### 2.6. Potentiometric Procedures

Emf measurements were monitored by immersing the potentiometric cell in the sample test solution, after equilibrium was reached. Stable readings of ±0.2 mV were achieved within 10–20 s. The cell was calibrated by decreasing the initial concentration of CA 15-3 with a suitable buffer. HEPES solution was used for this purpose; in this, aliquots ranging from 0.0025 to 4.5 mL of 1.0 × 10^−3^ mol/L HEPES were transferred into a 1.00 mL of CA 15-3 solution of 100 U/mL, prepared in same buffer.

The calibration data extracted from each calibration included limit of detection (LOD), slope, linear ranges (upper and lower limits), squared correlation coefficient and response time. The slope and correlation coefficient were calculated using excel from windows and extracted from the linear trend observed. The LOD was the concentration point corresponding to the intercept point between linear and non-linear behaviors in each calibration curve. The response time was the time necessary to reach a stable emf reading (±1 mV) within time. The upper and lower concentration limits corresponded to the limiting concentration intervals in which a linear or close-to-linear behavior was observed (defined by the correlation coefficient).

Serum samples were analyzed by spiking a synthetic serum solution (commercial Cormay serum, diluted 100 times) with an appropriate amount of CA 15-3 standard. The concentration of CA 15-3 was determined by using the analytical data of a control calibration curve in synthetic control serum, made previously.

### 2.7. Selectivity

The selectivity was assessed by means of potentiometric selectivity coefficients, determined by the mixed solutions method. For this purpose, a solution of a given interfering species was prepared in HEPES buffer, setting its concentration to the average level expected in the plasma. CEA (0.03 ng/mL) and IL-6 (1.66 pg/mL) were selected for this purpose. Such solution was used as background medium, to start a new calibration of the primary ion (CA 15-3). Having the calibration ready, the limit of detection (LOD) was estimated from it and compared to the LOD of a normal calibration (with no interfering species).

## 3. Results and Discussion

### 3.1. Synthesis of the Imprinted Material

The biomimetic material was obtained by electropolymerization. The monomer selected for this work was Py, largely used in electrochemical studies. The polymerization reaction was initiated and sustained by applying a potential that was sufficiently elevated to oxidize the monomer. In this condition, the neutral Py lost one electron, yielding a cation radical. The formation of the cation radical was perceptible in the recorded voltammogram, in which a sudden current increase occurred at +0.8 V (versus an AgCl/Ag reference electrode). Next, the dimerization of the cation radical occurred by binding to another unit of Py and losing 2H^+^. Such dimer was more easily oxidized than the original Py, due to its higher degree of conjugation [29].

In turn, the physical arrangement of the polymer nanostructures depended on the electrical conditions related to the electropolymerization. Considering previous studies on a stainless steel surface, the nanostructural arrangement of PPy could vary from nanobelts, to nanobricks or nanosheets, depending of the scan-rate of the CV [29,34]. For high scanning speeds (200 mV/s) the time allowed for deposition and dissolution of the polymeric network decreased, yielding a porous structure with a low level of crystallization, organized in multiple layers of nanosheets. In contrast, for decreasing scanning speeds, the time given for polymer growth was longer, resulting in denser structures of a compact layer of nanobelts. From the point of view of an imprinted material for a protein target, the best nanostructural arrangement of PPy seemed the nanobelt. This structural organization, in the shape of an extended tube, could allow a more efficient molding of the protein and the maintenance of the imprinted cavity after the removal of the target protein.

**Figure 1:**
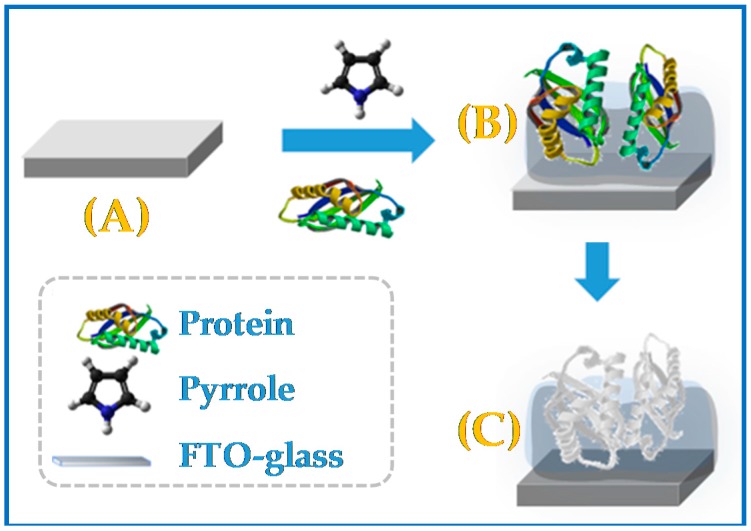
A schematic illustration for the molecular imprinting synthesis. (**A**) Fluorine doped tin oxide (FTO)-glass support. (**B**) Electropolymerization of Pyrrol in the presence of the target protein (CA 15-3). (**C**) Removal of the protein with ethanol.

Thus, the MIP material was obtained by polymerization of Py in the presence of CA 15-3 making use of electrochemical conditions that we would expected to lead to the production of PPy nanobelts (the formation of such nanostructures was not, however, confirmed by electronic microscopy). Moreover, the presence of CA 15-3 in the growing polymeric structure would have worked as an obstruction to the nanostructural alignment expected for the polymer molecules, resulting in deformities of the structure of the polymer. These deformities would correspond, therefore, to CA 15-3 proteins imprisoned within the polymeric matrix (Figure 1).

The imprinted material was obtained by scratching the dark dust formed on the glass support (a similar approach was made for the control NIP material). The scratching out of the polymeric film originated the exposure of entrapped proteins, due to the fracturing of the cavities in which these were entrapped. Removal of the protein from any exposed cavity happened by desorption with ethanol, which promoted denaturation of the peptide fragments.

The imprinted material was ready to use, after the extraction of non-reacted species or peptide residues that could be absorbed in the polymeric structure. These biomolecules were removed with an intensive wash of the polymer in pure ethanol first, and then in water. Finally, the solids were allowed to dry at 60 °C.

### 3.2. Surface Analysis of the Imprinted Materials

**Figure 2:**
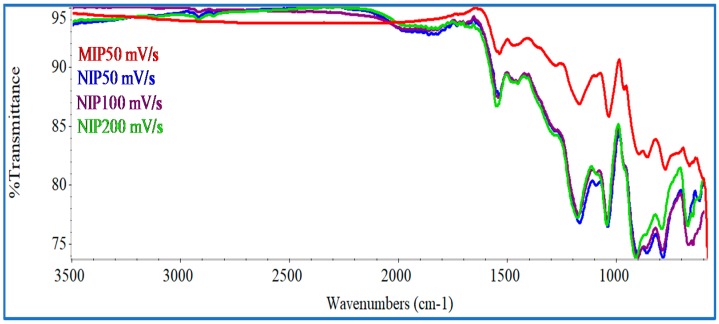
Fourier transform infrared spectroscopy (FTIR) spectra in full scale of the non-imprinted polymer (NIP) materials produced with different scan-rates (50, 100 and 200 mV/s).

FTIR analysis was conducted for NIP PPy materials obtained with scanning speeds of 50, 100 and 200 mV/s, and for MIP materials obtained with scanning speeds of 50 mV/s. The spectra obtained are shown Figure 2. Several relevant peaks were common among the MIP and the different NIP structures, mostly revealing the presence of PPy [35,36]. The bands at 1537.2 and 1454.1 cm^−1^ were related to the vibrational stretch of C=C and C–C bonding in Py, respectively. The peak at 1165.1 cm^−1^ was assigned for N–C stretching band, and 1036.8 cm^−1^ to the vibrational plane stretch of C–H bonding. The peak at 963.2 cm^−1^ was attributed to the vibrational deformation of the C=C bonding in the aromatic ring of Py. The adsorption at 860.6 cm^−1^ was assigned to the out of plan vibration of =C–H bonding. Finally, the adsorption bands at 780.9 and 665.3 cm^−1^ were assigned to deformation, in and out of the plane, of C–H bonding in Py units.

Overall, the FTIR spectra obtained in this work were very close to those reported with the stainless steel electrode for the same CV scanning speeds [29]. These spectra were similar along the presented wavenumber range, varying only in the relative intensity of the two peaks located within 600 and 800 cm^−1^.

**Figure 3:**
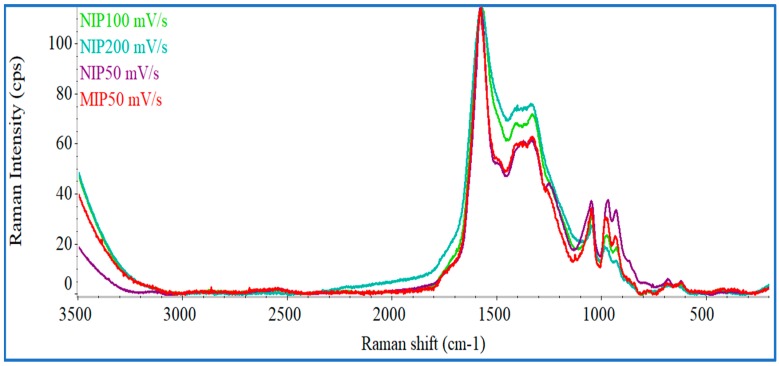
Raman spectra of NIP materials prepared with scan-rates of 50, 100 and 200 mV/s and the corresponding molecularly-imprinted polymers (MIPs) material with 50 mV/s.

Figure 3 shows the Raman spectra for all control materials (NIP, using CV scan-rates of 200, 100, or 50 mV/s) and MIP materials obtained with the scanning-rate of interest (50 mV/s). The overall NIP spectra corroborated well with several PPy Raman spectra presented in the literature [35,36]. The two highest picks at 1576.2 cm^−1^ (G band) and 1382.6 (D band) cm^−1^ are typical of PPy. The relative increase of Raman intensity (I) of these bands expressed as I_D_/I_G_ ratio reflected the increasing disorders present within in materials. For MIP and NIP materials obtained with the scanning-rate of 50 mV/s, the ratio was 0.84 and 0.85, respectively. These values are quite similar suggesting that the template removal from the polymeric matrix did not affect the structure of the polymer. The highest intensity peak in all spectra was associated to stretching modes of the C=C bonding present in PPy. The band at 1382.6 cm^−1^ was assigned to the stretching mode of pyrrole ring, and the band at 1047.7 cm^−1^ was associated to the in- and out-of-plane vibrational bonding of C–H and symmetrical reflection planes of the ring. The bands observed at 984.52, 683.10 and 634.12 cm^−1^ corresponded to the vibrational deformation of the Py ring [29,35,36].

In general, it was possible to observe that the spectral profile of the different materials was similar, differing mostly on the peaks centered at 1330–1380 cm^−1^, in which the Raman intensity increased with rising scanning-rate. In a general way, this region was related to the presence of carbon with sp^3^ hybridization, typically implying defects on a carbon matrix with resonance. In the present context, the results obtained may imply that the lower scanning speed promoted the reduction of the structural disorganization, having a more evident formation of structures with resonance (and therefore, sp^2^ hybridization). The differences between of the peaks centered at 1047.73 and 984.72 cm^−1^ also supported the great influence of the scanning speed upon the electropolymerization of Py.

**Figure 4:**
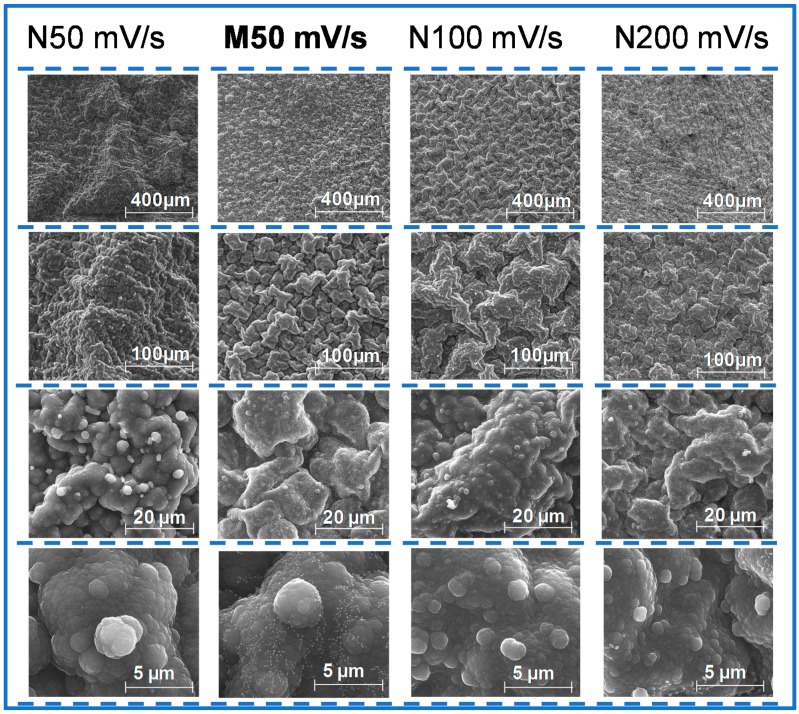
Scanning electron microscope (SEM) images of the PPy MIP materials prepared at a scan-rate of 50 mV/s and PPY NIP materials prepared at 50, 100 and 200 mV/s.

Figure 4 shows SEM analysis of NIP material deposited on FTO-glass with 50, 100 and 200 mV/s scan-rates. The images of lower magnification seem to be related to a different microstructural arrangement, which is indeed more or less consistent with the drawings presented in [29] for this purpose. However, higher magnification levels enter into a closer detail of the material and do not reflect such differential supra-organization. This is possibly related with fact that PPy was growing on an FTO-glass substrate, in a PBS medium. FTO-glasses are far from being a flat support, which would hinder the differentiation between such nanostructural arrangements. The composition of PBS had more salt concentration compared to a simple electrolyte as potassium nitrate. Concerning the MIP materials produced with 50 mV/s, also shown in Figure 4, it seemed that the final structure was more compact than the corresponding NIP material. This was an evidence that the protein interfered in the polymerization process and subsequently in the final nanostructure of the polymer [29]. Overall, SEM images are not conclusive regarding the observation of imprinted sites (as expect), giving an general idea about the morphological structure of each polymer obtained.

Overall, the combined FTIR, Raman and SEM data confirmed the polymerization of Py and the great impact of the CV scanning-rate, upon the nanostructural organization of the polymer being formed. The existence of changes when the protein is present also suggested that a successful protein imprinting process had taken place.

### 3.3. Analytical Features of the ISEs

The ability of the antibody-like material to interact with CA 15-3 was tested by applying these materials as selective ionophores in ISEs. For this purpose, the MIP powder was dispersed in a plasticizer agent, well dissolved in PVC, and the resulting polymeric membranes casted on a solid conductive support, made of graphite and epoxy resin [37]. Aiming to improve the CA 15-3 detection response, MIP sensors were also prepared with PClTPB and TOB, acting, respectively, as anionic and cationic lipophilic compounds. Usually, adding an ionic compound of lipophilic nature to the selective membranes reduces the electrical resistance, thereby increasing its ion-exchange ability. Control materials were also prepared, by replacing the MIP material by the NIP.

The overall composition of the prepared membranes is listed in Table 1. To evaluate the analytical behavior of each membrane, International Union of Pure and Applied Chemistry (IUPAC) recommendations for the evaluation of ion-selective electrodes were followed [38], whenever possible. In general, the lower limit of linear range (LLLR) was the minimum concentration displaying a linear behavior against the log concentration of CA 15-3; the corresponding upper limit (ULLR) was the highest concentration level after which the linear behavior was no longer observed; and the Limit of detection (LOD) was the concentration corresponding to the intercept point between linear and non-linear behaviors.

#### 3.3.1. Analytical Performance of the ISEs in Buffer

**Figure 5:**
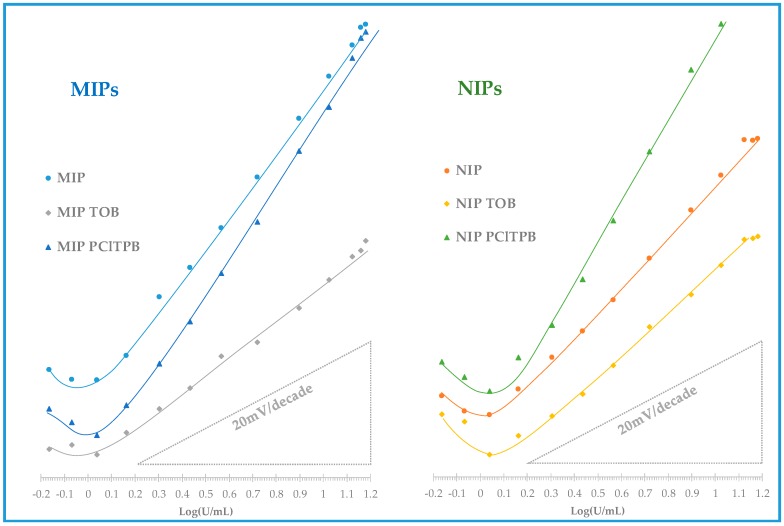
Potentiometric response of CA15-3 PVC membrane sensors with CA 15-3 standard solutions prepared in HEPES buffer, in pH 6.5.

As previously explained, the main analytical features of the prepared electrodes were assessed by evaluating the analytical data extracted from calibrations, according to IUPAC recommendations [38], first in buffer background medium. The values obtained concerned mostly LOD, LLLR, ULLR, time of response and the correlation coefficient and slope of the calibration curve (as shown in Table 2, corresponding to at least 3 repeated calibrations). The corresponding calibration curves are also shown in Figure 5.

In HEPES buffer, 1.0 × 10^−3^ mol/L, pH 6.5, all ISEs displayed positive slopes and thereby a cationic response (Table 2). However, this was the opposite behavior because the protein should be slightly negatively charged in this buffer, considering that its isoelectric point lies within 3 to 5.0. One possible explanation for this observation is that CA15-3 has many ionisable functional groups on its surface and may interact with the membrane from different sides. This means that the protein may approach the membrane from different sides, and affect the overall potential depending on the way this was occurring, which in turn would also depend from the ionic charge carrying each selective membrane.

As may be seen in Table 2, ISEs with the MIP material as ionophore displayed higher average slopes (44.5 mV/decade) than the corresponding NIP ionophore (35.5 mV/decade). These electrodes with MIP as ionophore and no additive also displayed a linear behavior for lower concentrations (1.44 U/mL) and lower LOD (1.07 U/mL) than the corresponding NIP sensors. Their liner range of response was also wider, up to a concentration of 13.2 U/mL.

The addition of an anionic lipophilic additive, PClTPB, aimed to improve the electrical conductivity of the membrane, thereby improving the sensitivity of the potentiometric response for a cationic species, within theoretical slopes. Herein, membranes with MIP + PClTPB and NIP + PClTPB displayed a linear response down to 1.51 and 2.04 U/mL, with detection limits of 1.51 and 1.58 U/mL, and slopes of 49.8 and 61.1 mV/decade, respectively (Table 2). Comparing to membranes without additive, the effect of the ionic additive was confirmed, with slopes increasing in 10%, in the case of MIP-based membranes and in 70% the case of the NIP-based membranes. Although higher sensitivity is typically better, the fact that the additive increased a lot the slope of the NIP material was a clear indication that the response was being dominated by the additive. Moreover, as this effect was less evident in the MIP membrane with PClTPB, it suggested that the MIP material was capable of imposing its response against such dominant effect of the additive. Overall, the effect of PClTPB did not improve clearly the response of the polymeric materials towards a more sensitive/selective response driven for CA 15-3, and the MIP material showed a higher discriminating ability against the target protein.

A cationic additive based on quaternary ammonium compound, TOB, was also added to the membranes. This additive may act as a cation excluder, which would contribute to improve the selectivity of the response. Membranes with this additive also showed a cationic response against the protein, but lower sensitivity and worse LOD and LLLR. The MIP+TOB and NIP + TOB sensors exhibited a linear response from 1.45 and 2.04 U/mL, with a detection limit of 1.18 and 1.18 U/mL and an average cationic slope of 25.5 and 30.8 mV/decade. In general, compared to sensors with no additive, the presence of TOB reduced the sensitivity of the response in the same concentration range. This effect was consistent with the presence of a cationic element within the membrane, which would contribute to diminishing the ion-exchange of a cationic species (as expressed by the prepared electrodes). As with the anionic additive, membranes with NIP materials displayed higher sensitivity, meaning that the behavior of the MIP was more affected by the additive, suggesting that it had a higher ability to discriminate CA 15-3 than the NIP. Overall, the effect of this additive was not particularly relevant, as the sensitivity was too low, compared to membranes without additive (only 57% average slopes in the case of the MIP materials and 87% in the case of NIP materials).

Overall, ISEs with MIP particles as electroactive materials without additive showed the best behavior comparing all ESIs. The corresponding membranes had the best LOD and wider linear range (Table 2, and Figure 5). Moreover, these MIP-based membranes had a higher slope (in 25%) than the corresponding NIP-based membranes, thereby confirming that the MIP had partially the ability to discriminate CA 15-3 by imprinted sites. All electrodes were kept in this study, particularly to observe if there was any impact of the additives upon selectivity.

#### 3.3.2. Stability and Time of Response

All calibrations were made after allowing an initial period of stabilization in the initial CA 15-3 100 U/mL solution prepared in buffer. After this, the necessary time to reach stability was always less than 60 s. For this purpose, the emf was considered stable when it changed within time ±1 mV.

To reuse the electrodes, washing periods in buffer were established between each calibration, aiming to remove bound proteins and set the potential to a “blank” level. For this purpose, after each calibration, the membranes were soaked in buffer, until reaching more or less the same emf as before the first calibration. This procedure improved the repeatability of the electrodes. Consecutive long-term calibrations showed very low potential drift.

The lifetime of the electrodes working on a daily basis (working days) was set in two weeks, as the typical analytical features were kept within of ±5% for this period.

#### 3.3.3. Selectivity of the Potentiometric Response

**Figure 6:**
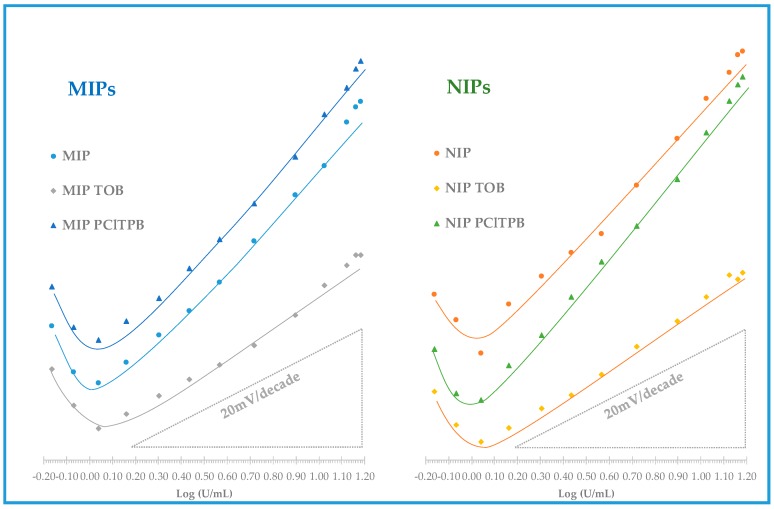
Potentiometric response of CA15-3 PVC membrane sensors with CA 15-3 standard solutions prepared in HEPES buffer, in pH 6.5, in the presence of a constant concentration of CEA.

**Figure 7:**
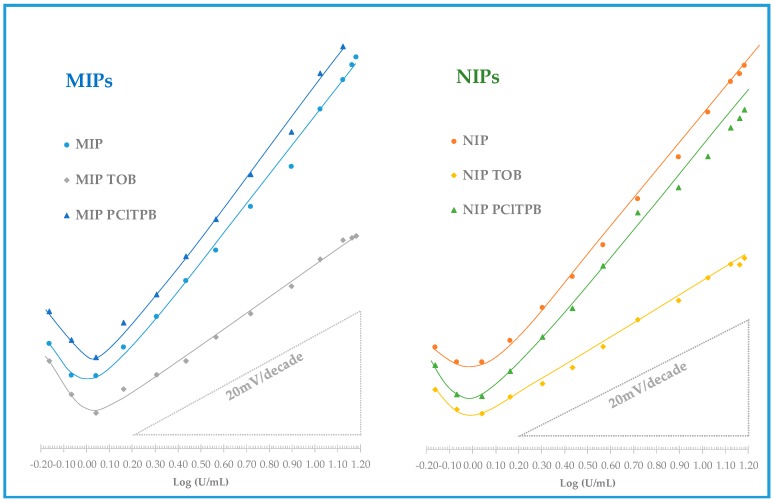
Potentiometric response of CA15-3 PVC membrane sensors with CA 15-3 standard solutions prepared in 4-(2-Hydroxyethyl)-1-piperazineethanesulfonic acid (HEPES) buffer, in pH 6.5, in the presence of a constant concentration of IL6.

The mixed solution method was employed to assess the selectivity of the electrodes, reporting the effect of each interfering species in terms of change in the analytical features, compared to calibrations in the absence of the interfering species. The interfering species selected for this study were CEA and IL-6, tested in their typical higher concentrations in serum, and assuming that their presence above these limits is unlikely. The values obtained are listed in Table 3 and Table 4 and the corresponding calibrations in Figure 6 and Figure 7.

In general, considering membranes having only MIP materials, there were no significant changes in terms of analytical features with or without interfering species. The % slope or % LOD deviation between these calibrations were less than 10%. The corresponding NIP membranes showed a higher impact, with 17% slope increase when CEA was present. The linear range of response was more or less the same for membranes containing MIP or NIP material.

In the presence of a given interfering species (CEA or IL-6), the membranes with lipophilic anionic additive has this time a similar behavior to the corresponding membranes without the additive: the concentration ranges of linear responses were the same and the slopes varied 0.4–6% (MIP material) or 1–9% (NIP material). This was not expected, but it seems that the anionic additive controlled to some extent the effect of these interfering species. Although this was a point in favor of these membranes, it was difficult to explain the much higher slopes obtained when the calibrating solutions contained only CA 15-3.

Regarding the effect of the cationic additive, the observed behavior confirmed the contribution of this compound as cationic excluder. In general, the slope of the MIP + TOB in buffer (25.5 mV/decade) was not significantly affected by the presence of CEA (23.1 mV/decade) or IL-6 (24.0 mV/decade). In turn, the NIP + TOB slope was more affected by the presence of interfering species, changing from 30.8 mV/decade in buffer to 21.7 mV/decade in the presence of CEA and to 22.2 mV/decade in the presence of IL-6. Thus, comparing the effect of the interfering species of membranes with TOB, it was clear that the additive contributed to improve the selectivity, but this was only observed when MIP materials were used as ionophore.

Overall, the electrodes displaying better behavior had MIP materials working as ionophore and required no additive to improve the response. The cationic additive had a positive impact upon selectivity but a negative impact upon sensitivity. In turn, the negative additive had a non-consistent behavior when calibrations with or without interfering species were compared.

### 3.4. Application of the Electrodes in CA 15-3 Assay

The analysis of spiked synthetic serum was considered to test a possible application for the CA 15-3 ISEs. For this purpose, the ISEs were first calibrated in a background of diluted serum. Cormay serum 100× diluted in buffer was used for this purpose. Cormay serum is a very complex matrix that really simulates the composition of human serum. Each spiked sample was analyzed immediately after calibration of the ISEs.

In general, the analytical features of all electrodes revealed lower slopes (Table 5), meaning that the diluted serum had a negative impact upon the sensitivity. However, the linear ranges remained more or less the same as those obtained in calibrations with buffer. Gathering all data obtained with all ISEs, the electrodes selected to carry on with the analysis of spiked serum were ISEs containing membranes with only MIP material as ionophore (no additive). The corresponding NIP materials were also tested for comparison purposes (to confirm if the imprinted materials would govern the analytical results obtained).

The diluted Cormay serum was spiked with three different levels of CA 15-3 concentration: 5.2, 2.7 and 1.5 U/mL. These concentrations were selected in agreement with the physiological levels of CA15-3 in human serum. The data obtained is shown in Table 6. In general, ISEs with MIP material yielded average recoveries of 97.2%, ranging from 96.3 to 97.7%. The relative error corresponding to this study varied ranged 3.7 to 2.3%. In turn, the NIP material showed a worse behavior, as shown in Table 6.

Overall, the analytical results in serum confirmed the precision and the accuracy of the data produced by the MIP-based electrodes, thereby confirming their suitability to be applied in the analysis of serum samples. The decreased sensitivity promoted by serum had no impact upon the analytical data generated.

## 4. Conclusions

The production of a novel antibody-like material by electropolymerizing Py has been found simple and effective. The resulting material exhibited a compatible behavior as ionophore in potentiometric transduction, aiming at the selective monitoring of CA 15-3 in serum. The presence of a lipophilic additive within the selective was found unnecessary to have suitable analytical features by means of a polymeric-based material acting as ionophore. The molecular imprinting had a significant contribution to the observed response, as confirmed by the analytical features and the analysis of spiked serum samples.

Compared to previous methods in Table 7, the present work showed some advantageous features. The cost of the equipment and electrodes is very low, the electrode bodies are easy to build, stable and have a long lifetime (several years) and electrodes with sensing membranes last for 2 weeks (may be reused several times in this period, requiring calibration prior to sample analysis). Overall, the proposed method was simple, affordable, and accurate, suggesting further advantages in screening applications.

## Figures and Tables

**Table 1:** Membrane composition of CA15-3 sensors.

Component	Weight (mg)					
	ISE I	ISE II	ISE III	ISE IV	ISE V	ISE VI
	MIP	NIP	MIP + TOB	NIP + TOB	MIP + PClTPB	NIP + PClTPB
MIP	2.34	—	2.34	—	2.34	—
NIP	—	2.34	—	2.34	—	2.34
TOB	—	—	0.32	0.32	—	—
PClTPB	—	—	—	—	0.32	0.32
oNFOE	29.30	29.30	29.30	29.30	29.30	29.30
PVC	14.10	14.10	14.10	14.10	14.10	14.10

**Table 2:** Main analytical performance of the ISEs evaluated in 1.0 × 10^−3^ mol/L HEPES, pH 6.5.

Analytical Feature	MIP	NIP	MIP + TOB	NIP + TOB	MIP + PClTPB	NIP + PClTPB
Slope (mV/decade)	44.5 ± 1.8	35.5 ± 8.6	25.5 ± 0.9	30.8 ± 1.4	49.8 ± 1.5	61.1 ± 6.0
R^2^ (n = 3)	0.9981	0.9969	0.9941	0.9979	0.9981	0.999
LOD (U/mL)	1.07	1.15	1.18	1.18	1.51	1.58
LLLR (U/mL)	1.44	2.04	1.45	2.04	1.51	2.04
ULLR (U/mL)	13.2	10.5	13.2	13.2	10.5	13.2
Response time (s)	60 s	60 s	60 s	60 s	60 s	60 s

LOD: limit of detection; LLLR: Lower Limit of Linear Range; ULLR: Upper Limit of Linear Range.

**Table 3:** Main analytical performances of the ISEs in the presence of interfering specie CEA under the specified concentrations, in pH 6.5.

Analytical Feature	MIP	NIP	MIP + TOB	NIP + TOB	MIP + PClTPB	NIP + PClTPB
Slope (mV/decade)	40.6 ± 1.4	35.2 ± 3.1	23.1 ± 1.4	21.7 ± 0.9	40.8 ± 1.4	38.5 ± 1.7
R^2^ (n = 3)	0.9902	0.9909	0.9871	0.9918	0.9927	0.997
LOD (U/mL)	1.17	1.10	1.07	1.02	1.20	1.02
LLLR (U/mL)	1.44	1.10	1.45	1.10	1.10	1.10
ULLR (U/mL)	13.2	13.2	10.5	13.2	13.2	13.2
Response time (s)	60 s	60 s	60 s	60 s	60 s	60 s

LOD: limit of detection; LLLR: Lower Limit of Linear Range; ULLR: Upper Limit of Linear Range.

**Table 4:** Main analytical features of the ISEs in the presence of interfering specie IL-6 under the specified concentrations, in pH 6.5.

Analytical Feature	MIP	NIP	MIP + TOB	NIP + TOB	MIP + PClTPB	NIP + PClTPB
Slope (mV/decade)	43.1 ± 2.2	41.5 ± 1.8	24.0 ± 1.7	22.2 ± 2.1	45.6 ± 1.5	40.0 ± 3.5
R^2^ (n = 3)	0.9926	0.9947	0.9928	0.9958	0.9945	0.9947
LOD (U/mL)	1.02	1.02	1.05	1.02	1.02	1.02
LLLR (U/mL)	1.10	1.10	1.10	1.10	1.10	1.10
ULLR (U/mL)	13.2	13.2	10.5	13.2	13.2	13.2
Response time (s)	60 s	60 s	60 s	60 s	60 s	60 s

LOD: limit of detection; LLLR: Lower Limit of Linear Range; ULLR: Upper Limit of Linear Range.

**Table 5:** Main analytical features of the ISEs in Cormay Serum HM, at pH 6.5.

Analytical Feature	MIP	NIP	MIP + TOB	NIP + TOB	MIP + PClTPB	NIP + PClTPB
Slope (mV/decade)	32.4 ± 2.7	27.4 ± 3.4	14.0 ± 1.6	8.1 ± 6.3	34.2 ± 1.3	35.8 ± 0.6
R^2^ (n = 3)	0.9937	0.9808	0.9751	0.9119	0.996	0.994
LOD (U/mL)	1.07	1.15	1.18	1.18	1.51	1.48
LLLR (U/mL)	1.45	2.04	1.45	2.04	1.51	2.04
ULLR (U/mL)	13.2	10.5	13.2	13.2	10.5	13.2
Response time (s)	60 s	60 s	60 s	60 s	60 s	60 s

LOD: limit of detection; LLLR: Lower Limit of Linear Range; ULLR: Upper Limit of Linear Range.

**Table 6:** Analytical response of the ISEs to spiked serum samples, after previous calibration in Cormay serum HM as background medium.

Sample	CA 15-3 (U/mL)	MIP		NIP	
		Error (%)	Recovery (%)	Error (%)	Recovery (%)
1	5.24	2.8 ± 1.0	97.2	7.3 ± 2.0	92.7
2	2.72	3.7 ± 1.8	96.3	6.0 ± 0.6	94.0
3	1.45	2.3 ± 0.3	97.7	10.8 ± 6.4	89.2

**Table 7:** Comparison of methods for detection of CA15-3.

Technique	Linear Range	LOD	Comments	Ref.
Electrochemiluminescence	0.1 to 100 U/mL	0.1 µU/mL	High price. Needs 4 °C storage. Complex assembly.	[1]
Electrochemiluminescence	0.05 to 120 U/mL	0.014 U/mL	High price. Needs 4 °C storage. Complex assembly.	[2]
Electrochemiluminescence	0.1 to 120 U/mL	0.030 U/mL	High price. Need 4 °C storage. Electrodepositing AuNPs.	[4]
Electrochemistry	0.002 to 40 U/mL	3 × 10^−4^ U/mL	Modified graphene sheets. Needs 4 °C storage.	[5]
Electrochemistry	0.1 to 160 U/mL	0.04 U/mL	Higher price. Need 4 °C storage. Very complex sensor.	[7]
Chemiluminescence	0.3 to 20 U/mL	0.2 U/mL	High price.	[6]
Surface-enhanced Raman spectroscopy (SERS)	0.1 to 500 U/mL	0.13 U/mL	Very high price the equipment. Very complex.	[3]
Optofluidic ring resonator sensor	1 to 200 U/mL	1 U/mL	Higher price. Need CO_2_ lasers. Very complex sensor.	[8]
Potentiometry (Ion-selective electrodes)	1.44 to 13.2 U/mL	1.072 U/mL	Very low cost. Simple and quick. Higher life time.	This work
